# *Bacillus anthracis* Poly-γ-D-Glutamate Capsule Inhibits Opsonic Phagocytosis by Impeding Complement Activation

**DOI:** 10.3389/fimmu.2020.00462

**Published:** 2020-03-31

**Authors:** Shikhar Sharma, Rakesh Bhatnagar, Deepak Gaur

**Affiliations:** ^1^Laboratory of Malaria and Vaccine Research, School of Biotechnology, Jawaharlal Nehru University, New Delhi, India; ^2^Molecular Biology and Genetic Engineering Laboratory, School of Biotechnology, Jawaharlal Nehru University, New Delhi, India

**Keywords:** *Bacillus anthracis*, poly-γ-D-glutamate, capsule, complement, phagocytosis, opsonization

## Abstract

*Bacillus anthracis* poly-γ-D-glutamic acid (PGA) capsule is an essential virulent factor that helps the bacterial pathogen to escape host immunity. Like other encapsulated bacterial species, the *B. anthracis* capsule may also inhibit complement-mediated clearance and ensure bacterial survival in the host. Previous reports suggest that *B. anthracis* spore proteins inhibit complement activation. However, the mechanism through which the *B. anthracis* capsule imparts a survival advantage to the active bacteria has not been demonstrated till date. Thus, to evaluate the role of the PGA capsule in evading host immunity, we have undertaken the present head-to-head comparative study of the phagocytosis and complement activation of non-encapsulated and encapsulated *B. anthracis* strains. The encapsulated virulent strain exhibited resistance toward complement-dependent and complement-independent bacterial phagocytosis by human macrophages. The non-encapsulated Sterne strain was highly susceptible to phagocytosis by THP-1 macrophages, after incubation with normal human serum (NHS), heat-inactivated serum, and serum-free media, thus indicating that the capsule inhibited both complement-dependent and complement-independent opsonic phagocytosis. An increased binding of C3b and its subsequent activation to C3c and C3dg, which functionally act as potent opsonins, were observed with the non-encapsulated Sterne strain compared with the encapsulated strain. Other known mediators of complement fixation, IgG, C-reactive protein (CRP), and serum amyloid P component (SAP), also bound more prominently with the non-encapsulated Sterne strain. Studies with complement pathway-specific, component-deficient serum demonstrated that the classical pathway was primarily involved in mediating C3b binding on the non-encapsulated bacteria. Both strains equally bound the complement regulatory proteins C4BP and factor H. Importantly, we demonstrated that the negative charge of the PGA capsule was responsible for the differential binding of the complement proteins between the non-encapsulated and encapsulated strains. At lower pH closer to the isoelectric point of PGA, the neutralization of the negative charge was associated with an increased binding of C3b and IgG with the encapsulated *B. anthracis* strain. Overall, our data have demonstrated that the *B. anthracis* capsule inhibits complement fixation and opsonization resulting in reduced phagocytosis by macrophages, thus allowing the bacterial pathogen to evade host immunity.

## Introduction

*Bacillus anthracis* is the causative agent of a one of the most lethal zoonotic diseases, anthrax, which affects both humans and livestock. It is endemic in developing countries, whereas its outbreaks in the developed world are primarily through intentional means (1, 2). The deliberate release of *B. anthracis* as a biological weapon remains a great threat throughout the world (3). Major characteristics that establish *B. anthracis* as a potent natural pathogen and a bioterrorism agent include its extremely high virulence and immune evasion ability (4–6). Thus, it is imperative to understand the pathophysiology of *B. anthracis* infection and develop effective intervention strategies to both prevent and cure anthrax. Apart from exotoxins that critically suppress the host immune system (7, 8), the essential virulent factor for *B. anthracis* is its extracellular capsule (9, 10). Unlike many bacterial species that have a polysaccharide capsule, the *B. anthracis* capsule is poly-γ-D-glutamic acid (PGA) in nature, which is believed to provide a survival advantage to the bacteria by deceiving the host immune surveillance (11). Previous reports have suggested that encapsulated strains of several bacterial pathogens such as *Neisseria meningitidis* and *Streptococcus pneumoniae* are resistant to phagocytosis relative to their non-encapsulated counterparts (12–14). The importance of *B. anthracis* capsule as a virulent determinant is supported by the observations that all the clinical isolates causing progressive anthrax are encapsulated strains and a loss of capsule by either enzymatic degradation or genetic mutations reduces the virulence of the bacteria (10).

The complement system is known to be an integral part of innate immunity, which works in coordination with phagocytes and facilitates the clearance of the pathogen. It is thus crucial in coordinating immune surveillance, inflammation, and clearance of cell debris (15). The complement system also interacts with the adaptive immune system and facilitates its activation through downstream signaling pathways (16). Consequently, this builds a selection pressure on the pathogens to evolve strategies to counteract complement-mediated clearance (17). To ensure their survival within the host, many parasites and pathogens have developed immune evasion strategies to circumvent complement mediated killing or lysis. These include the expression of cellular components and proteins on the pathogen surface that inhibit complement activation (18), blocking, or destabilization of the C3 convertase (19, 20); recruitment of host complement regulatory proteins on the pathogen surface (21, 22); and complement degradation by host plasminogen (23, 24). Previous reports have suggested that the *B. anthracis* spore coat protein BclA has the propensity to bind with complement factor H (FH) and human serum plasminogen (24, 25), which leads to C3 breakdown and imparts anti-opsonic properties to the spore. Considering the common characteristics of complement evasion and a higher survival rate exhibited by encapsulated bacteria compared with their non-encapsulated counterparts, it is conceivable that the *B. anthracis* PGA capsule may also exhibit complement antagonistic properties (11).

The complement system involves around 50 proteins ranging from plasma proteins to membrane-associated proteins. Complement proteins are pattern recognition molecules (PRMs) that bind with distinctive pathogen surface markers (26, 27). The complement system has three functional pathways termed as classical, alternative, and mannan-binding lectin (MBL) pathways (15), which are differentiated on the basis of their stimuli required to initiate each cascade. The classical pathway initiates through the binding of the C1 complex with the antibodies tethered to pathogen-associated molecular patterns (PAMPs) on the pathogen surface. This in turn stimulates the C1 allied protease to cleave the complement components, C2 and C4, to form the protease complex C4b2a, which acts as a C3 convertase and cleaves the C3 molecule into smaller fragments, C3a and C3b. The C3 cleavage produces structural changes in C3b such as the unmasking of thioester linkages that facilitate the binding of C3b with hydroxyl and amino groups on the surface of the invading pathogen (28). Surface-associated C3b generated through the classical pathway also feeds into the alternative pathway as C3b has the affinity for binding with the plasma protein factor B (FB), to form a pro-convertase C3bB (15). C3bB undergoes cleavage by another plasma protein factor D (FD) into a functional C3 convertase C3bBb that further cleaves more C3 and forms an amplification loop for C3b deposition on the pathogen surface (15). Both the classical and alternative pathways of complement activation contribute toward deposition of C3b (29). The increasing density of bound C3b either leads to the formation of the “membrane attack complex” (MAC) or initiates effector operations for pathogen clearance (15). C3b has a binding specificity for the complement receptor CR1 (CD35) on immune cells and promotes opsonization (15). C3b interaction with CD35 mediates the degradation of C3b into iC3b and a smaller fragment C3f or iC3b that gets further degraded to C3dg and C3c. Whereas the C3c fragment is a fluid-phase protein, iC3b and C3dg remain surface bound and mediate opsonization. iC3b binds with CR3 (CD11b) and CR4 (CD11c) molecules present on phagocytic cells and augments complement-mediated phagocytosis. iC3b also binds with CR2 (CD21), a receptor expressed on B-cells, which leads to reduction of the activation threshold of B-cells and promotes generation of memory cells (15, 26, 27).

The exterior location of the capsule makes it a primary host immune modulating structure of pathogenic bacteria. Non-encapsulated bacterial strains of *B. anthracis* have been demonstrated to be more prone to phagocytosis than encapsulated strains (30), but the differential extent of complement protein fixation on their surface has not yet been demonstrated. The role of the PGA capsule in resisting phagocytosis by either preventing complement activation or IgG binding with *B. anthracis* remains unknown. Thus, considering the essential role of complement activation in pathogen clearance, a comprehensive understanding of the complement interaction with the bacterial capsule of *B. anthracis* and its contribution in virulence is of immense significance.

In the present communication, we have conducted a systematic head-to-head comparative study for the binding of complement protein C3b, complement mediators such as C-reactive protein (CRP) (31), serum amyloid P component (SAP) (32), and human IgG (33) on the bacterial cell surface of virulent encapsulated strains and avirulent, non-encapsulated strains of *B. anthracis*. Our studies have demonstrated the mechanisms underlying the differential extent of opsonic phagocytosis and complement activation of encapsulated and non-encapsulated strains of *B. anthracis*.

## Materials and Methods

### Bacterial Strains and Culture Conditions

The *Bacillus anthracis* encapsulated virulent strain was obtained from the Defense Research & Development Establishment (DRDE) Gwalior, Madhya Pradesh (34). The well-characterized, laboratory-adapted, non-encapsulated Sterne (34F2) strain has been described previously (35). Both *B. anthracis* strains were cultured at 37°C with 5% CO_2_ in brain heart infusion (BHI) broth (Himedia) with 0.8% sodium bicarbonate to an OD_600_ of 0.6 that correlates to a bacterial count of ≈10^8^ colony-forming units (CFU)/ml (11). Aliquots were prepared of 10^8^ CFU in 20% glycerol and stored at −80°C. Growth conditions of both the encapsulated, virulent and non-encapsulated, avirulent strains were similar.

### PCR Amplification With Capsule Specific Primers

*B. anthracis* strains were cultured in BHI agar plates. Single colonies were suspended in 20 μl of nuclease free water (Invitrogen) and heated at 100°C for 10 min. Boiled samples were centrifuged at 10,000 × g for 5 min. Supernatant was used as template to amplify pXO2 plasmid-specific genes *capA* and *acpB* using specific primers (Table S1). The same supernatant was also used to amplify a fragment of the *lef* gene. The primer sequences used for the amplification of the pXO1- and pXO2-specific genes are described in Table S1.

### Macrophage Phagocytosis

Phagocytosis was investigated using the well-established CFU count-based bacterial internalization assay (36). The human monocytic leukemic cell line, THP-1 (37), was cultured in Roswell Park Memorial Institute (RPMI) media (Sigma, Cat. No. R8758) supplemented with 10% fetal bovine serum (Thermo, Cat. No. 10270106) and penicillin–streptomycin solution (Thermo, Cat. No. 15140122). THP-1 cells were seeded in a six-well plate with a density of 10^6^ cells per well. THP-1 cells incubated at 37°C under 5% CO_2_ with 10 nM of phorbol-12-myristate-13-acetate (PMA) (Sigma-Aldrich P1585) differentiated into macrophages. The differentiated and adhered cells were infected with 10^7^ bacteria/well, establishing a multiplicity of infection (MOI) of 1:10. Infections were carried in RPMI media supplemented with normal human serum (NHS) (Merck S1-100ML) or heat-treated human serum (ΔNHS) or no human serum. Three different human serum concentrations (12.5, 25, and 50%) were used to establish infection and for analyzing dose-dependent phagocytosis. For heat inactivation, serum was heated at 56°C for 30 min (38), to denature heat-labile complement proteins while keeping serum antibodies intact. Cytochalasin D-treated THP-1 cells were used as negative control for phagocytosis (39). To inhibit phagocytosis, THP-1 cells were incubated with 5 μM of cytochalasin D (Sigma) for 30 min at room temperature. Bacterial cells resuspended in RPMI were added to the differentiated THP-1 cells. Infected THP-1 cells were incubated for 2 h at 37°C with 5% CO_2_. Post incubation, the non-internalized bacteria in RPMI media were collected, and the THP-1 cells were washed 10 times with sterile phosphate-buffered saline (PBS). THP-1 cells were lysed with 0.02% sodium dodecyl sulfate (SDS) in sterile PBS and plated on BHI agar. The number of viable bacteria either remaining in the culture media or internalized by macrophages were calculated and expressed as percentage of the number of bacterial CFU used as the inoculum for each experiment.

### Flow Cytometry-Based Binding Assay

The deposition of the C3b complement protein and mediator proteins CRP and SAP was analyzed through a fluorescence-activated cell sorting (FACS)-based binding assay. For measuring the binding of serum immune components with the *B. anthracis* strains, bacterial cells were fixed using 4% paraformaldehyde (Sigma, Cat. No. P6148) and incubated in NHS for 30 min at 37°C (40). Cells were pelleted and washed thrice with sterile PBS supplemented with 0.05% Tween 20 (0.05% PBST). Serum-incubated bacterial cells probed with secondary antibodies were used as negative control. C3b deposition on the bacterial surface was assessed by the flow cytometry assay using mouse anti-human C3b monoclonal antibodies (Thermo Fisher, Cat. No. MA1-70054) and goat anti-mouse Alexa Fluor 488 (Thermo Fisher, Cat. No. A32723). Dose-dependent C3b binding was analyzed by incubating bacteria with a concentration gradient (10, 25, 50, and 100%) of NHS (Merck). CRP and SAP binding on the bacterial surface was assessed using mouse anti-human CRP (Thermo Fisher, Cat. No. MA5-17061) and mouse anti-human SAP monoclonal antibodies (Thermo Fisher, Cat. No. LF-MA0160). Rabbit anti-human C4BP polyclonal antibodies (Thermo Fisher, Cat. No. PA5-42001) and mouse anti-human FH monoclonal antibodies (BioLegend, Cat. No. 518401) were used for analyzing the binding of C4BP and FH, respectively. The role of individual complement pathways was analyzed using human serum deficient in pathway-specific molecules. C3b binding was performed as described above with C1q-deficient human serum (AssayPro, Cat. No. D710110) and factor D-deficient human serum (AssayPro, Cat. No. D770110). C5-deficient human serum (AssayPro, Cat. No. D510110) was used as positive control for both pathways. For setting a threshold value of the non-specific binding of the fluorescent-tagged secondary antibodies, bacteria incubated in sterile PBS with secondary antibodies were taken as a control. The percentage of bacteria bound with a particular serum component was represented by the mean fluorescence index (MFI) that has been widely used to combine the binding intensity and fraction of affected bacteria (12, 41).

### Immunoblot Assay for Human IgG and C3b Binding and Activation

Binding of C3b and IgG was analyzed by immunoblot; 10^8^ CFU were suspended in sterile PBS and incubated with increasing concentrations of NHS (0, 10, 25, and 50%) for 30 min at 37°C with constant shaking. For investigating C3b and IgG binding on the encapsulated strain at a pH range 2.4–7.4, the bacterial cells were suspended in sodium phosphate–citrate buffers ranging from pH 2.4 to 7.4 and incubated with 10% NHS or purified human IgG. Cells were pelleted by centrifugation at 10,000 × g for 10 min. Supernatants were removed, and cell pellets were washed thrice with sterile 0.05% PBST. Bacterial pellets were suspended in sterile PBS, and the lysates were prepared in a 2.5% SDS-based buffer. Complement proteins are heat-labile proteins (42). C3b was detected in bacterial lysates ran on a 10% SDS–polyacrylamide gel electrophoresis (SDS-PAGE) without heating under non-reducing conditions (without adding 2-mercaptoethanol), transferred to nitrocellulose membrane (MDI), and probed with mouse anti-human C3b monoclonal antibodies (Thermo Fisher, Cat. No. MA1-70054) followed with horseradish peroxidase (HRP)-conjugated goat anti-mouse secondary antibodies (Sigma, Cat. No. A4416). The serum-incubated bacterial cell lysates were also used to detect bound IgG. Bacterial lysate samples were prepared by adding 10% of 2-mercaptoethanol and heating at 95°C for 15 min. Lysates were separated on 12% SDS-PAGE and transferred to nitrocellulose membranes (MDI). Bound IgG was probed with goat anti-human IgG antibodies conjugated with HRP (Sigma, Cat. No. A8667). Activated C3b gets processed to yield a heat-stable fragment, C3c, in the fluid phase, as it does not remain surface bound. Reducing and non-reducing SDS-PAGE samples were prepared from supernatants of bacteria incubated with 10% NHS in PBS for 30 min. Samples were separated by 10% SDS-PAGE, transferred to nitrocellulose membranes (MDI), and probed with rabbit anti-human C3 polyclonal Ab (Thermo Fisher, Cat. No. PA5-21349) followed with the secondary HRP-conjugated, goat anti-rabbit antibodies (Sigma, Cat. No. A6154). The immunoblots were developed by enhanced chemiluminescence using SuperSignal West Pico Chemiluminescent Substrate (Thermo Fisher, Cat. No. 34577). Pre-stained protein ladders (GeneDireX, Cat. No. PM007-0500) were used as standards for the immunoblots. For each immunoblot, 1% NHS was used as positive control. As a loading control for the C3b and serum IgG binding experiments, the bacterial cell lysate samples used in the immunoblot assays were analyzed by SDS-PAGE and stained with Coomassie brilliant blue G-250 (Himedia).

### Statistical Analysis

The results are reported as mean ± SE post data preparation and statistical analysis. The results were compared between both bacterial strains by the Student unpaired *t*-test. Statistically significant differences between the groups are highlighted by the following denotations: ^*^ for *P*-value < 0.05, ^**^ for *P*-value < 0.01, and ^***^ for *P*-value < 0.001.

## Results

### Confirmation of the Poly-γ-D-Glutamate Capsule-Encoding pXO2 Plasmid in the Encapsulated *Bacillus anthracis* Strains

The *Bacillus anthracis* non-encapsulated strain Sterne (34F2) has previously been well characterized and reported to be non-virulent in mouse model studies (35). The encapsulated *B. anthracis* strain used in our study is a clinical isolate obtained from an infected carcass at DRDE (Gwalior, MP) and has been demonstrated to be virulent in mice (34). The presence of the toxin-encoding (pXO1) and capsule-encoding (pXO2) plasmids were confirmed by PCR using pXO1 and pXO2 specific primers (Figure S1). Both the *B. anthracis* strains are isogenic with the exception of the presence of capsule-encoding pXO2 plasmid in the virulent strain. *CapA* is a gene present in capsule biosynthesis operon (*capBCAED*). The *AcpB* gene is present in the pXO2 plasmid downstream to the capsule operon. The pXO2 genes (*CapA* and *AcpB*) were amplified by PCR from the encapsulated strain, whereas no amplification was detected from the non-encapsulated strain. These results confirmed that the virulent strain selected was positive for pXO2-encoded capsule genes. However, the pXO1 gene (*lef* ) encoding the lethal factor was detected in both the encapsulated and non-capsulated strains.

### *Bacillus anthracis* Poly-γ-D-Glutamate Capsule Inhibited Complement-Dependent and Complement-Independent Macrophage Phagocytosis

**Figure 1:**
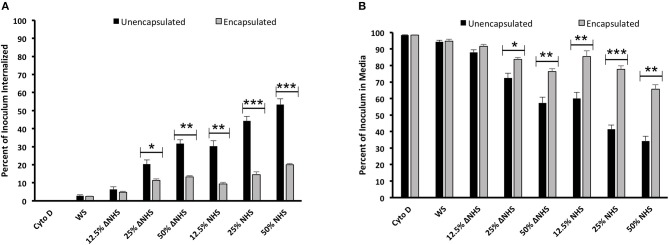
Differential phagocytosis of encapsulated and non-encapsulated strains of *Bacillus anthracis* active bacteria by human monocyte-derived macrophages. THP-1 macrophage cells were infected with *B. anthracis* in media supplemented with different concentrations of normal human serum (NHS; 12.5–50%) and heat-treated human serum (ΔNHS, 12.5–50%). **(A)** Internalized surviving bacteria obtained from lysed macrophages post-phagocytosis represented as percent of *B. anthracis* inoculum. **(B)** Percent of *B. anthracis* inoculum (non-internalized) surviving in the media post incubation with THP-1 macrophages. Black and gray bars represent non-encapsulated and encapsulated strains, respectively. Cytochalasin D (Cyto D)-treated THP-1 cells that have lost their phagocytic ability were considered as negative control. Each bar represents the mean of three independent experiments. Error bars represent standard error of the mean. WS, media without serum. Statistical significance is highlighted by the following denotations: * for *P*-value < 0.05, ** for *P*-value < 0.01, and *** for *P*-value < 0.001.

Infection of *B. anthracis* encapsulated and non-encapsulated strains was established with human monocyte-derived macrophages (THP-1); and the infectivity was measured based on CFU counts (Figure 1). The infection of THP-1 macrophage cells with *B. anthracis* strains was analyzed in RPMI media supplemented with either NHS or heat-treated human serum (ΔNHS) or media devoid of NHS (WS). The heat-treated human serum would not comprise of active complement proteins and thus allows a comparison of the contribution of the complement system in phagocytosis in relation to other serum components (including IgG). Infected THP-1 macrophages were lysed, and the released *B. anthracis* bacteria were measured by their CFU counts (Figure 1A). Similarly, the non-internalized *B. anthracis* bacteria were measured by the CFU counts obtained by culturing the media supernatant obtained post infection (Figure 1B). In order to ascertain that the released *B. anthracis* was primarily from infected macrophages, the THP-1 cells were treated with cytochalasin D (Cyto D) as a control prior to *B. anthracis* infection. Cyto D treatment inhibits actin polymerization, further impairing the phagocytic ability of the THP-1 macrophages (43, 44). No CFU counts were observed from the lysed Cyto D treated THP-1 macrophages (Figures 1A,B), suggesting that there were no residual bacteria that could account for the CFU counts.

Bacterial internalization and phagocytosis of both the encapsulated and non-encapsulated *B. anthracis* strains were analyzed under varying concentrations of NHS and ΔNHS (12.5, 25, and 50%) (Figure 1A). A significant increase was observed in the internalization of the non-encapsulated *B. anthracis* Sterne strain (Figure 1A), compared with the encapsulated strain. Phagocytosis of the non-encapsulated Sterne strains by the THP-1 cells was dose dependent, as percent internalization increased proportionately to the increase in serum concentration of both ΔNHS (6 ± 1.47% CFU at 12.5% ΔNHS; 20 ± 2.33% CFU at 25% ΔNHS; 31.6 ± 3.6% at 50% ΔNHS) and NHS (30 ± 2.96% CFU at 12.5% NHS; 44 ± 2.40% CFU at 25% NHS; 53.3 ± 5.5% at 50% NHS) (Figure 1A). The lower levels of phagocytic internalization observed in the heat-treated human serum (ΔNHS) suggest that the phagocytosis is dependent on the complement proteins (Figure 1A). The complement-independent phagocytosis observed with heat-treated human serum (ΔNHS) is based on the activity of other serum components including IgG. A substantially higher level of phagocytosis was observed with NHS (Figure 1A) where, in addition to the IgG, the complement system is functional.

In comparison, the percent internalization of the encapsulated *B. anthracis* was observed to be significantly lower under both conditions of NHS (9.4 ± 0.93% at 12.5% NHS; 14.5 ± 1.68% CFU at 25% NHS; 20 ± 0.75% at 50% NHS) and ΔNHS (4.8 ± 0.4% at 12.5% ΔNHS; 11.26 ± 0.92% CFU at 25% ΔNHS; 13.23 ± 0.79% at 50% ΔNHS) (Figure 1A). Thus, the encapsulated virulent strain exhibited resistance to both complement-dependent and complement-independent phagocytosis, further implying that the capsule was responsible for inhibiting both complement-dependent and complement-independent macrophage phagocytosis. The differential levels of phagocytosis or internalization were also measured by analyzing the number of non-internalized *B. anthracis* bacteria remaining in the culture supernatant (Figure 1B). The CFU counts observed after culturing the media supernatant under the two different human sera showed a dose-dependent decrease of the non-encapsulated Sterne strain with an increased concentration of the NHS or ΔNHS consistent with the fact that the number of bacteria in the culture supernatant would decrease under conditions of increased phagocytosis or internalization by the THP-1 macrophage cells (Figure 1B). However, no such decrease in CFU counts was observed with the encapsulated virulent strain consistent with their lower internalization by the THP-1 macrophage cells, and thus, the number of bacteria in the culture supernatant would largely remain unaltered (Figure 1B).

Thus, the complement-dependent or complement-independent phagocytosis of the *B. anthracis* bacteria by the macrophages was differentially higher for the non-encapsulated strains compared with the encapsulated strains.

### Poly-γ-D-Glutamate Capsule Inhibited C3b Deposition on *Bacillus anthracis*

**Figure 2:**
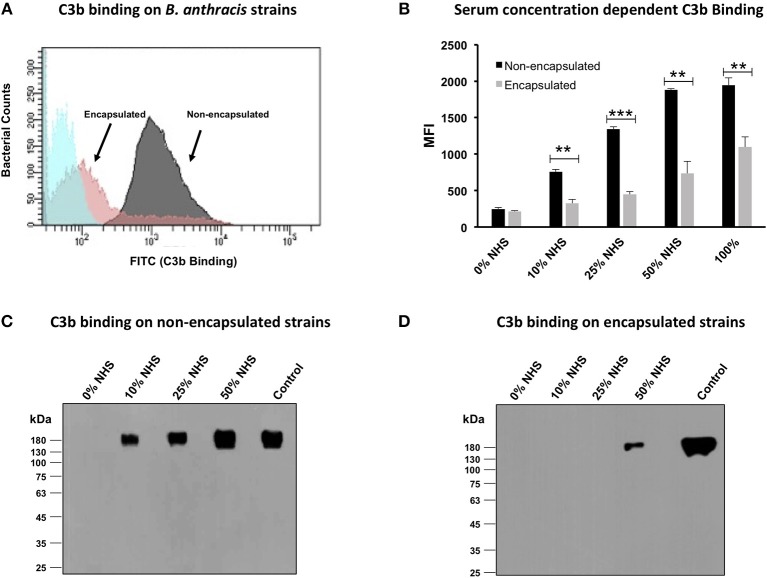
C3b deposition on encapsulated and non-encapsulated strains of *Bacillus anthracis*. **(A)** Flow cytometry histogram of C3b deposition on encapsulated and non-encapsulated *B. anthracis* strains incubated in 10% human serum. Gray and pink shading indicates non-encapsulated and encapsulated bacteria, respectively. Blue shading indicates bacteria incubated in phosphate-buffered saline (PBS) alone. **(B)** Mean fluorescence index (MFI) of serum concentration-dependent C3b deposition on encapsulated and non-encapsulated bacteria. Black and gray bars represent non-encapsulated and encapsulated strains, respectively. The scatter plots for the corresponding histograms are represented in Figure S3. **(C,D)** Immunoblot assays to detect serum concentration-dependent C3b deposition on non-encapsulated **(C)** and encapsulated strains **(D)**; 1% normal human serum was used as positive control. Each data point represents the mean of three independent experiments. Error bars represent standard error of the mean. Statistical significance is highlighted by the following denotations: ** for *P*-value < 0.01, and *** for *P*-value < 0.001.

C3b is a complement protein that is centrally involved in the activation of all the three complement pathways: classical, alternative, and MBL. Thus, in order to analyze the effect of the capsule on the activation of the complement pathways, we investigated the binding of C3b with the encapsulated and non-encapsulated *B. anthracis* strains, which was analyzed by both FACS and immunoblotting assays. Both approaches confirmed a marked increase in C3b binding with the non-encapsulated Sterne strain compared with the encapsulated *B. anthracis* strain (Figure 2). The flow cytometry histogram (Figure 2A) clearly depicts a stronger binding of C3b with the non-encapsulated Sterne strain with a higher MFI than with the virulent encapsulated strain in 10% NHS. Importantly, C3b binding was dose dependent, as the MFI for C3b binding with both *B. anthracis* strains increased proportionately with the concomitant increase in serum concentration (Figure 2B), suggesting a specific binding interaction. The MFI for C3b binding observed with the encapsulated bacteria at the four concentrations of NHS (10, 25, 50, and 100%) increased from 326 ± 52.2 to 1,095 ± 141.9, whereas the MFI for C3b binding with non-encapsulated bacteria was observed at a significantly higher range of 658 ± 127 to 1,943 ± 100.5 (Figure 2B). The MFI was reflective in a higher 79.2% of the non-encapsulated *B. anthracis* strain being positive for C3b binding in comparison with only 22% of the encapsulated Sterne strain (Figure S3). Thus, we clearly demonstrated that C3b binding was significantly reduced on encapsulated bacteria compared with the non-encapsulated bacterial strain.

Outer capsules of pathogens have a hairy kind of structure that has been reported to impede access of antibodies against surface-bound proteins (10, 45), thus raising the possibility that the *B. anthracis* capsule might also be restricting the antibodies to access the C3b that may be reflected in the FACS-based C3b binding assays. Thus, in order to validate the lower C3b binding observed with the encapsulated bacteria, the C3b binding was also analyzed by immunoblots in which lysates of *B. anthracis* bacteria after post-incubation with NHS were probed with using specific anti-human C3b monoclonal antibodies under non-reducing conditions. C3b is a human complement protein that does not exist in *B. anthracis*, and thus, any signal detected in the immunoblot assay would reflect the C3b protein bound to the outer bacterial surface. C3b is a 180-kDa protein composed of two polypeptide chains bound through a disulfide linkage, which was thus detected by immunoblotting under non-reducing conditions. Binding of the 180-kDa C3b molecule was observed with the non-encapsulated *B. anthracis* in a dose-dependent manner, as the intensity of the bound protein increased with increasing concentrations of the NHS: 0 to 50% NHS (Figure 2C). In comparison, C3b binding with the encapsulated *B. anthracis* virulent strain was observed at a significantly lower degree. No C3b binding was observed with the encapsulated strain at the serum concentrations of 0–25% (Figure 2D). The 180-kDa C3b protein was detected only at a high concentration (50%) of NHS (Figure 2D). An equal loading of the bacterial lysate samples used in the immunoblot assays was confirmed by SDS-PAGE (Figure S2). Thus, both the FACS and immunoblot assay approaches consistently confirmed the differential binding of the complement protein C3b between the encapsulated and non-encapsulated strains of *B. anthracis*.

### Poly-γ-D-Glutamate Capsule Inhibited the Downstream Activation of C3b to C3c and C3dg

After binding with the target cells, complement protein C3b gets activated and undergoes downstream processing to form several smaller molecules including C3d, which remains attached with the pathogen surface, and C3c, which is released in the culture supernatant. We have demonstrated that C3b binds more efficiently to the non-encapsulated *B. anthracis* strain compared with the encapsulated strain. In order to study the downstream processing of the bound C3b protein with the encapsulated and non-encapsulated strains, the formation of the thermostable, fluid-phase protein C3c was analyzed by immunoblot assay using the culture supernatant.

**Figure 3:**
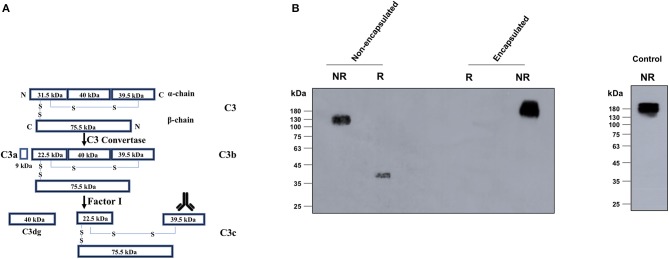
Detection of the C3c processed fragment with encapsulated and non-encapsulated strains of *Bacillus anthracis*. **(A)** Schematic representation of the C3 processing during complement fixation depicting the cleavage of C3 into C3b, C3c, and C3d. C3b remains surface bound, whereas C3c is released in the fluid-phase media. **(B)** Non-encapsulated and encapsulated strains of *B. anthracis* were incubated in 10% normal human serum and C3c (138 kDa) detected in the supernatants by immunoblot assays using specific antibodies against the 40-kDa C-terminal fragment of alpha chain of C3c (marked by the antibody image in **A**). C3c formation was only observed with the non-encapsulated Sterne strain. The 140-kDa C3c (alpha and beta chains) protein and 40-kDa C3c (alpha chain C-terminus) were detected under non-reducing and reducing conditions, respectively, with the non-encapsulated *B. anthracis* Sterne strain. The unprocessed 189-kDa C3 protein from human serum was detected in the supernatants of the encapsulated virulent strain consistent with the same detected in the 1% normal human serum (positive control). R, reducing; NR, non-reducing.

*B. anthracis* encapsulated and non-encapsulated strains were incubated in 10% NHS, and the C3c protein was observed in the fluid-phase culture supernatant. Immunoblots of the culture supernatants were probed with C3 polyclonal antibodies specific to the N-terminal 40-kDa fragment of the C3c alpha chain (Figure 3). C3c protein was detected under both reducing (40-kDa) and non-reducing (137-kDa) conditions from serum incubated with the non-encapsulated *B. anthracis* strain (Figure 3). However, no C3c formation was observed in serum incubated with the encapsulated virulent strain (Figure 3), thus suggesting that the low levels of C3b protein detected to bind with the capsule do not undergo further downstream processing.

### C3b Deposition on Non-encapsulated *Bacillus anthracis* Strain Is Primarily More Dependent on the Classical Pathway

Complement fixation occurs primarily through two pathways: classical or alternative. To understand the contribution of the classical or alternative pathway in the C3b deposition on non-encapsulated strain, C3b binding was analyzed by FACS in the presence of two human sera, each deficient in one major component of the two pathways: C1q-deficient serum (essential component for initiation of classical pathway) and factor D-deficient serum (essential component of alternative pathway). Human serum deficient in the C5 protein (downstream complement cascade component not involved directly in C3b binding) was used as a positive control.

**Figure 4:**
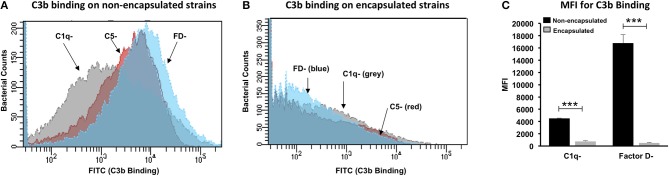
Contributory role of classical and alternative pathways in C3b deposition on *Bacillus anthracis*. Flow cytometry histogram for C3b deposition on non-encapsulated **(A)** and encapsulated **(B)** *B. anthracis* strains incubated in 10% human serum deficient in complement component C1q (C1q–), factor D (FD–), and C5 (C5–). Gray, red, and blue histogram shading represents C3b deposition in C1q–, C5–, and FD– deficient sera, respectively. **(C)** C3b binding represented as mean fluorescence index (MFI) observed with non-encapsulated (black bars) and encapsulated (gray bars) *B. anthracis* strains incubated in C1q and FD sera. Each bar represents the mean of three independent experiments. Error bars represent standard error of the mean. Statistical significance is highlighted by the following denotations: *** for *P*-value < 0.001.

A significant reduction in C3b deposition was observed on encapsulated bacteria incubated with C1q-deficient sera (Figure 4), thus suggesting that the classical pathway had a major role in binding of the C3b complement protein. Factor D-deficient sera had no impact on C3b binding (Figure 4), suggesting that the alternative pathway had a low contribution toward fixation of the C3b protein. Thus, our results demonstrate that the classical pathway is responsible for mediating C3b binding and deposition on the *B. anthracis* non-encapsulated Sterne strain. The encapsulated bacterial strain incubated with serum deficient in either C1q or factor D or C5 did not exhibit binding with C3b (Figure 4), implying that the poly-γ-D-glutamate capsule played a key role in impeding complement deposition and subsequent complement-dependent phagocytosis (Figure 4).

### Poly-γ-D-Glutamate Capsule Inhibited Binding of Mediators of the Classical Complement Pathway

We have demonstrated the C3b complement protein binds more effectively to the non-encapsulated *B. anthracis* compared with the encapsulated strain and that the binding is primarily dependent on the classical complement pathway. Our results suggest that the encapsulated *B. anthracis* strain has specifically devised mechanisms to inhibit the classical complement pathway. Thus, we further investigated the interaction of the classical pathway mediators such as IgG, CRP, and SAP with the *B. anthracis* strains (33, 46, 47).

**Figure 5:**
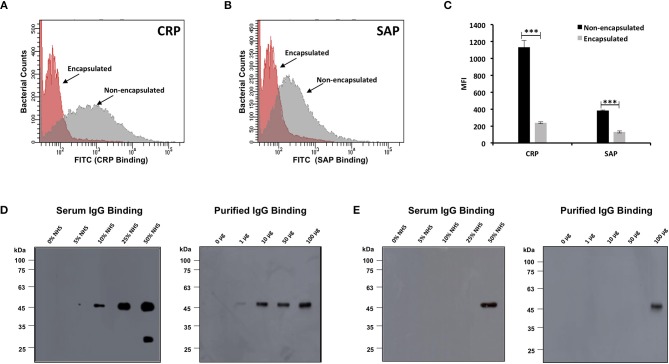
Binding of complement mediators with encapsulated and non-encapsulated strains of *Bacillus anthracis*. Flow cytometry histogram for binding of pentraxins C-reactive protein (CRP) **(A)** and serum amyloid P component (SAP) **(B)** on *B. anthracis* strains incubated in 10% human serum. Gray and red shading indicates non-encapsulated and encapsulated bacteria, respectively. **(C)** CRP and SAP binding represented as mean fluorescence index (MFI) observed with non-encapsulated (black bars) and encapsulated bacteria (gray bars). Each bar represents the mean of three independent experiments. Error bars represent standard error of the mean. The scatter plots for the corresponding histograms are represented in Figures S4, S5. **(D,E)** Immunoblot assay for detection of human serum IgG and purified human IgG binding with *B. anthracis* non-encapsulated strains **(D)** and encapsulated strains **(E)** incubated with increasing concentration of normal human serum (5–50%) and purified human IgG (1–100 μg/ml); 1% normal human serum and 10 μg/ml of IgG was used as positive control. Statistical significance is highlighted by the following denotations: *** for *P*-value < 0.001.

Encapsulated and non-encapsulated *B. anthracis* strains incubated in 10% NHS were analyzed for CRP and SAP interaction by flow cytometry. The FACS-based assay demonstrated that in comparison with encapsulated strain, non-encapsulated strain exhibited a stronger binding affinity for CRP (Figures 5A,C) and SAP (Figures 5B,C). A higher percent of the non-encapsulated *B. anthracis* bacterial population were observed to bind with CRP (54.6%) (Figure S4) and SAP (26.6%) (Figure S5). In comparison, only 4.8% (Figure S4) and 4.6% (Figure S5) of the encapsulated bacteria bound with CRP and SAP, respectively.

Complement regulatory proteins, C4BP and FH, have been reported to degrade the C3 convertase of the classical pathway (C4b2a) and alternate pathway (C3bBb), respectively (15, 48). C3 convertase is responsible for cleaving C3 in to C3a and C3b. Thus, a difference in the binding of C4BP and/or FH between the encapsulated and non-encapsulated strains of *B. anthracis* could also contribute toward the difference in deposition of the C3b opsonin and in turn lower complement activation with encapsulated *B. anthracis* strains. We analyzed the binding of C4BP and FH with both encapsulated and non-encapsulated strains of *B. anthracis* but could not observe any significant difference that could explain the above phenotype (Figure S6). Thus, both complement regulators (C4BP and FH) exhibited an equivalent binding with the encapsulated and non-encapsulated strains of *B. anthracis* (Figure S6).

Interaction of serum IgG with both *B. anthracis* strains was analyzed by immunoblot assay. Binding of human serum IgG and purified human IgG (gamma globulins) with the non-encapsulated *B. anthracis* was observed in a dose-dependent manner, as the intensity of binding increased with the increasing concentration of NHS (0 to 50% NHS) and purified total IgG (1–100 μg/ml) (Figure 5D). In comparison, serum and purified IgG binding with the encapsulated *B. anthracis* virulent strain was observed at a significantly lower degree. No IgG binding was observed with the encapsulated strain when the bacteria were incubated at serum concentrations of 0–25% NHS and 1–50 μg/ml. Bound IgG was detected only in a high 50% concentration of NHS and 100 μg/ml of purified IgG (Figure 5E). IgG is an integral component of the classical pathway, and our results confirm that the classical pathway is more crucial for complement activation on surface of non-encapsulated *B. anthracis*.

### The Negative Charge of Poly-γ-D-Glutamate Capsule Inhibits the Binding of C3b and IgG

We have demonstrated that the encapsulated *B. anthracis* strain resists the binding of complement proteins, C3b and IgG, in comparison with the non-encapsulated strains. The significantly lower binding of serum opsonins on the encapsulated *B. anthracis* strain is associated with their reduced phagocytosis. The encapsulated and non-encapsulated *B. anthracis* strains are isogenic with the exception of the poly-γ-D-glutamate capsule. *B. anthracis* capsule is a polymer of D-glutamic acid, which has an isoelectric point of 3.22 and hence imparts a net negative charge to the bacterial outer capsule and surface at physiological pH. We thus analyzed the role of the negative charge of the poly-γ-D-glutamate capsule in inhibiting the binding of human IgG and C3b on the surface of encapsulated *B. anthracis* strain.

**Figure 6:**
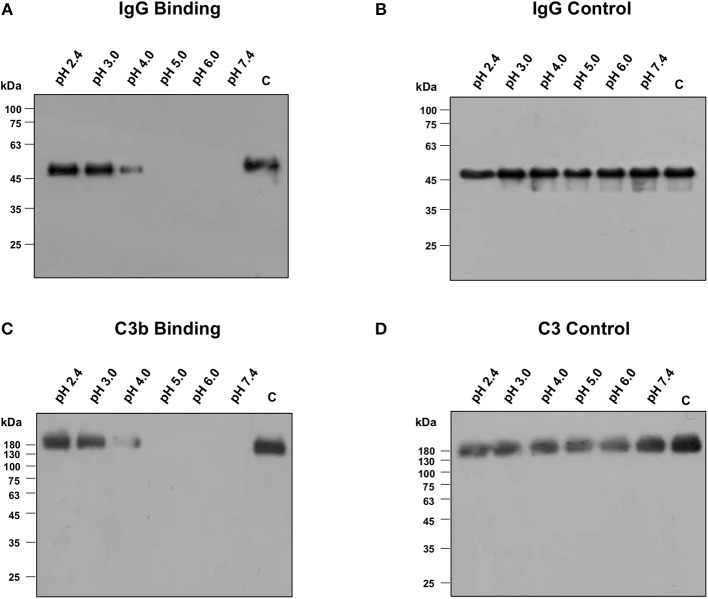
Binding of IgG and C3b with encapsulated strain of *Bacillus anthracis* incubated at different pH buffers ranging from 2.4 to 7.4, supplemented with 10% normal human serum or 50 μg/ml of purified human IgG. **(A)** Immunoblot assay for the detection of purified IgG binding on encapsulated strain of *B. anthracis*. IgG binding was observed at pH closer to the isoelectric point of poly-γ-D-glutamate (pI 3.2). **(B)** Purified IgG was stable at different pH buffers as detected by immunoblot. **(C)** Immunoblot assay for the detection of human C3b binding on encapsulated strain of *B. anthracis*. C3b binding was also observed at pH closer to the isoelectric point of poly-γ-D-glutamate. **(D)** Normal human serum (NHS) was incubated at different pH buffers and C3 was observed to be stable at low pH as analyzed by immunoblot.

To investigate the role of the negative charge, the encapsulated *B. anthracis* strain was incubated at six different pH buffers ranging from 2.4, 3.0, 4.0, 5.0, 6.0, to 7.4 supplemented with NHS. Previous binding studies in our current submission were all conducted at physiological pH 7.4 where the encapsulated *B. anthracis* strains exhibited significantly poor binding with IgG and C3b. Consistent with these results, we observed no binding of purified IgG (Figure 6A) or C3b (Figure 6C) at pH 7.4 where the poly-γ-D-glutamate capsule would be negatively charged. However, as the encapsulated bacteria were exposed to lower pH closer to the isoelectric point where the negative charge would be neutralized, the binding of purified IgG and C3b was detected at pH of 2.4, 3.0, and 4.0 (Figures 6A,C). The stability of the purified IgG and serum C3b molecules at the varying pH was analyzed to check for any potential degradation or precipitation (Figures 6B,D). Both molecules were observed to be stable and present in a soluble form at the different pH buffers (Figures 6B,D).

Our results have demonstrated the binding of IgG and C3b with the encapsulated strains at pH closer to the isoelectric point of poly-γ-D-glutamate where the capsule's negative charge would be neutralized, thus confirming the role of the negative charge of the poly-γ-D-glutamate capsule in inhibiting the binding of complement proteins on the encapsulated *B. anthracis* strains.

## Discussion

The complement system is an integral component of the innate immune system and acts as a bridge between innate and acquired immunity. It is composed of a large number of distinct plasma proteins that are activated to opsonize pathogens and induce inflammatory responses to counter infections. Therefore, many pathogens have developed mechanisms to evade complement-mediated clearance. It has been previously reported that complement depletion in C57BL/6 mice by genetic manipulation renders them sensitive to infection by the avirulent *Bacillus anthracis* Sterne strain (49). Thus, complement fixation is a crucial immune effector mechanism that contributes in the defense and clearance of the anthrax pathogen. *B. anthracis* spores have also devised strategies for the inactivation and binding inhibition of the C3b complement protein. The spore coat protein BclA binds to both human serum plasminogen and complement regulator FH that induces C3 degradation and subsequently inhibits complement-mediated clearance (24, 25). Other encapsulated bacteria such as *Streptococcus pneumoniae* and *Neisseria meningitidis*, which have a polysaccharide capsule, have also been reported to be more resistant to complement binding and opsonic phagocytosis compared with their non-encapsulated counterparts. The *B. anthracis* capsule has a distinct molecular composition as compared with other encapsulated bacterial species (4). Thus, it is important to better understand the specific interactions of the PGA capsule with the active immune effector molecules. Studies on the interaction of the immune effector molecules such as complement proteins and its mediators with the encapsulated and non-encapsulated *B. anthracis* strains are lacking. Hence, elucidating the molecular mechanisms underlying the immune evasion characteristics of the encapsulated *B. anthracis* bacteria would significantly help in designing more effective anthrax intervention strategies.

Phagocytic cells are the primary pathogen-clearing centers of the immune system, and complement proteins are crucial mediators of opsonic phagocytosis of the pathogen (27). The present study has aimed to understand the role of the poly-γ-D-glutamate capsule in immune evasion of the active bacilli of *B. anthracis* from macrophage-dependent opsonic phagocytosis. Therefore, we conducted a head-to-head systematic comparison of the phagocytic susceptibility and complement fixation ability of encapsulated virulent and non-encapsulated, avirulent strains of *B. anthracis*.

We first investigated bacterial internalization of both encapsulated and non-encapsulated strains of *B. anthracis* by the THP-1 in a complement-dependent and complement-independent manner. Our results have demonstrated an increased internalization of the non-encapsulated Sterne strain opsonized with NHS, compared with the encapsulated virulent bacterial strain, which was associated with a higher C3b deposition on the bacterial surface and its downstream activation. Complement-inactivated serum produced a significant decrease in bacterial internalization, confirming the essential role of complement fixation in the phagocytosis of *B. anthracis* active bacteria. The use of cytochalasin D treatment that impedes phagocytic ability of macrophages confirmed that the difference in internalization of encapsulated and non-encapsulated strains by the THP-1 macrophages was primarily attributed to the presence and absence of the bacterial capsule. However, complement-independent phagocytic internalization of the Sterne strain was also observed with heat-treated serum, which could be attributed to human IgG acting as an opsonin. The encapsulated strains demonstrated a greater resistance against phagocytosis under heat-treated serum conditions, consistent with the same phenomenon observed under NHS, suggesting that the capsule also inhibited IgG-dependent phagocytosis.

In order to understand the contribution of different complement pathways in enhanced C3b binding on the Sterne strain, C3b binding was analyzed with either C1q-deficient serum or factor D-deficient serum. A significant decrease in C3b deposition was observed only in C1q-deficient serum, implying that the classical pathway was primarily responsible for complement fixation of the active bacteria of the *B. anthracis* Sterne strain. Our data are consistent with previous reports that have shown that C3b fixation on *B. anthracis* spores enhances phagocytosis by human alveolar macrophages (50). C3b deposition on the *B. anthracis* spore surface was initiated primarily through the classical pathway, as the spore protein BclA has the propensity to bind with the serum complement regulator FH and to inhibit the alternative pathway (25).

We have thus demonstrated the lack of efficient opsonic phagocytosis of encapsulated *B. anthracis* bacteria, which is attributed to the inhibition of complement activation. Based on our extensive literature search (17, 18, 48, 51, 52), such a resistance to complement activation may be explained by one of the following mechanisms: (i) lack of binding of activating molecules (e.g., IgG, SAP, and CRP) on the bacterial surface; (ii) recruitment of complement regulators on the bacterial surface (e.g., C4BP for the classical pathway or FH for the alternative pathway); and (iii) presence of a bacterial protein or factor that can regulate complement or a cell surface protease that can cleave surface-bound C3b. In the present report, we have systematically evaluated these potential mechanisms of impeding complement activation and subsequently leading to an inhibition of opsonic phagocytosis.

IgG, SAP, and CRP have been established as activators of the classical complement pathway by facilitating the initial binding of C1q on the pathogen surface (33, 46, 47). Thus, the binding of these molecules has been reported to promote complement activation through the classical pathway. We have clearly demonstrated a lower binding of all three molecules, IgG (serum IgG and purified gamma globulins), SAP, and CRP with the encapsulated strain of *B. anthracis* compared with the non-encapsulated strain. Thus, the capsule of *B. anthracis* active bacteria inhibits the binding of IgG, CRP, and SAP, which is associated with reduced C3b/iC3b deposition on the bacterial surface. The observation of naïve human serum, exhibiting a higher binding affinity of IgG and other soluble PRMs such as SAP with the non-encapsulated Sterne strain than the encapsulated strain, suggests that a significant fraction of naturally acquired PRMs recognize sub-capsular antigens such as cell wall peptidoglycan or cell surface protein antigens (53). It appears that the capsule masks the sub-capsular antigens from human serum IgG and complement proteins, which has been consistently observed in our complement-dependent and complement-independent phagocytosis experiments. Therefore, this difference in binding of the key activators of the classical complement pathway could contribute as an underlying mechanism toward the subsequent lower complement activation on the encapsulated *B. anthracis* strains and in turn resulting in poor opsonic phagocytosis.

The recruitment of complement regulators on the bacterial surface (e.g., C4BP for the classical pathway or FH for the alternative pathway) has been reported for several pathogens such as *Streptococcus pyogenes* and *N. meningitidis* (17). C4BP and FH have been well characterized to inhibit the classical pathway C3 convertase (C4b2a) and alternate pathway C3 convertase (C3bBb), respectively. The C3 convertase is responsible for the cleavage of cleaving C3 in to C3a and C3b, a key downstream step in the complement activation pathways. Thus, a difference in the binding of C4BP and/or FH between the encapsulated and non-encapsulated strains of *B. anthracis* could explain the difference in deposition of the C3b opsonin and in turn lower complement activation with encapsulated *B. anthracis* strains. However, we could not observe any difference in the binding of C4BP and FH with both encapsulated and non-encapsulated strains of *B. anthracis*, which could explain the differential complement activation and opsonic phagocytosis.

It has been reported that several bacterial pathogens secrete extracellular proteases that regulate complement or cleave surface-bound C3. For example, *Staphylococcus aureus* and *Pseudomonas aeruginosa* secrete the C3 cleaving proteases, aureolysin and PaAP, respectively (54, 55). However, such a factor apart from the outer poly-γ-D-glutamate capsule playing a role in *B. anthracis* appears highly unlikely, as both the encapsulated and non-encapsulated *B. anthracis* strains that exhibit a significant difference in opsonic phagocytosis and complement activation have been reported to be identical in all respects except for the capsule encoded by the pXO2 plasmid (6). Thus, apart from the outer poly-γ-D-glutamate capsule, the encapsulated *B. anthracis* strains do not possess any extra protease or protein that are absent in non-encapsulated strains, which could explain the difference in complement activation. Hence, the poly-γ-D-glutamate capsule is the key determinant of complement inactivation on the surface of encapsulated *B. anthracis* strains that forms the basis of reduced opsonic phagocytosis.

To elucidate the underlying mechanism through which the encapsulated *B. anthracis* strain inhibits complement activation, we considered the potential role of the negative charge of the poly-γ-D-glutamate capsule. Poly-γ-D-glutamate has an isoelectric point of 3.22. Thus, at physiological pH 7.4, the poly-γ-D-glutamate capsule imparts a negative charge to the encapsulated bacteria. We investigated whether the negative charge had a role in inhibiting the binding of C3b and IgG on the encapsulated *B. anthracis*. In order to neutralize the charge, we exposed the encapsulated *B. anthracis* bacteria to a pH range of 2.4–7.4 and analyzed the C3b and IgG binding on the encapsulated bacteria. No binding of IgG or C3b was observed at pH 7.4 at which the poly-γ-D-glutamate capsule would be negatively charged. However, at a lower pH range of 2.4–4.0, closer to the isoelectric point of 3.22, the poly-γ-D-glutamate capsule would lose its negative charge; and this charge neutralization was associated with a binding of both serum IgG and C3b. We also confirmed that at the pH range of 2.4–7.4, the C3b and IgG were stable and detected in a soluble form, thus suggesting that no degradation or precipitation had occurred at lower or higher pH that could be reflected in our assay. Importantly, both C3b and IgG exhibited a functional binding activity at the lower pH where the negative charge of the poly-γ-D-glutamate capsule was neutralized.

Our data are consistent with previous studies where the negative charge of the cell surface has been reported to be critical in inhibiting the binding of antibodies and complement proteins on their surface (56). Encapsulated bacteria such as *N. meningitidis* possess a polysaccharide capsule composed of sialic acids that impart a negative charge to the bacterial surface and result in inhibition of IgG and complement binding on their surface (57). Enzymatic removal of sialic acid by neuraminidase renders the bacteria more prone to the binding of IgG and complement (57). Our results are consistent with those of previous studies, which have demonstrated that on cleavage of the poly-γ-D-glutamate capsule by enzymatic treatment, the encapsulated *B. anthracis* strain is rendered more sensitive to opsonic phagocytosis (11).

In summary, our study has demonstrated that the poly-γ-D-glutamate capsule suppressed complement fixation on encapsulated *B. anthracis*. The unique capsule of *B. anthracis* imparted a survival advantage to the encapsulated bacteria from human macrophages by inhibiting both complement-dependent and complement-independent phagocytosis. Encapsulated strains of *B. anthracis* exhibited a significantly lower C3b binding and activation on the bacterial surface. The presence of the capsule also restricted the binding of IgG, CRP, and SAP with the *B. anthracis* surface and eventually inhibited the classical complement pathway. Thus, the capsule impaired both complement-mediated and IgG-mediated opsonic phagocytosis. The negative charge of the poly-γ-D-glutamate capsule was demonstrated to be the underlying mechanism responsible for inhibiting complement binding and activation. The classical pathway was observed to play a primary and crucial role in complement fixation on the surface of non-encapsulated *B. anthracis* Sterne strain. Our results are consistent with previous reports with other pathogens that have also demonstrated that the capsule inhibits complement binding and activation on their surface (12, 14). *S. pneumoniae* and *N. meningitidis* polysaccharide capsules composed of sialic acids have been demonstrated to inhibit both the classical complement pathway and IgG-mediated opsonic phagocytosis (14).

To the best of our knowledge, ours is the first study to report the role of the poly-γ-D-glutamate capsule in mediating the differential complement fixation between encapsulated and non-encapsulated strains of the deadly pathogen, *B. anthracis*. Our study has elucidated some important aspects of the molecular mechanisms through which the PGA capsule mediates host immune evasion, and encapsulated *B. anthracis* strains establish an active infection. The combined effect of *B. anthracis* endotoxins and PGA capsule significantly contributes in evading the active bacteria from the effector arms of host immunity responsible for pathogen clearance.

Hence, we have clearly demonstrated that the poly-γ-D-glutamate capsule of *B. anthracis* is a key virulence factor that promotes the early establishment of active infection by evading the innate immune system. The PGA capsule released from *B. anthracis* has also been reported to play an active role in downstream steps of pathogenesis (58). Taken together, our study has further highlighted the key role of the PGA capsule in anthrax pathogenesis and further suggested it as a potent target for the development of novel intervention strategies to counter anthrax infections. Our study sets the stage for further investigations on the precise molecular mechanisms through the PGA capsule impedes the innate immune effector arms especially the binding of complement proteins, antibodies, and accessory proteins leading to phagocytic evasion. An in-depth understanding of these underlying mechanisms will enable the development of potent antibiotics and antimicrobials to counter this deadly infection.

## Data Availability Statement

All datasets generated for this study are included in the article/Supplementary Material.

## Author Contributions

SS and DG designed the study. SS conducted the experiments. DG supervised the study. RB provided key reagents and facilities. SS, RB, and DG analyzed the results and wrote the manuscript.

### Conflict of Interest

The authors declare that the research was conducted in the absence of any commercial or financial relationships that could be construed as a potential conflict of interest.
