# Supplementary material for: Bacillus anthracis Poly-γ-D-Glutamate Capsule Inhibits Opsonic Phagocytosis by Impeding Complement Activation
Source: Front Immunol. 2020 Mar 31;11:462. doi: 10.3389/fimmu.2020.00462 (PMC7138205; doi:10.3389/fimmu.2020.00462)
Supplement: Supplementary file 1 [file Presentation_1.pdf]

Supplementary Information

Bacillus anthracis Poly-γ-D-Glutamate Capsule Inhibits Opsonic Phagocytosis by Impeding Complement Activation

Shikhar Sharma, Rakesh Bhatnagar, Deepak Gaur*

School of Biotechnology, Jawaharlal Nehru University, New Delhi, India 110067

*Corresponding Author

Please address correspondence at the following address:

Professor Deepak Gaur
School of Biotechnology
Jawaharlal Nehru University
New Mehrauli Road,
New Delhi INDIA 110067
Tel: +91-11-26704012 / 26742616
+91-11-26738892
E-mail: deepakgaur189@gmail.com; deepakgaur@mail.jnu.ac.in

Supplementary Table

Table S1: List of primers used for amplifying pXO1 and pXO2 specific genes

Primers	Primer Sequence
**CapA Forward Primer**	TCAAGTTGTTGTCTCCACTGATACTTG
**CapA Reverse Primer**	GATAGTGCACTTGTGCAATATCATTTAC
**AcpB Forward Primer**	GTATATTGGTGTAATCGCGTTCTGTAGGG
**AcpB Reverse Primer**	CTGTTGAAGAAATTGCAAAAGTTACAATG
**Lef Forward Primer**	TTGGACTGAACATATGGCGGGCGGTCATGGT
**Lef Reverse Primer**	TCAGTAGGATCCGGATAGATTTATTTCTTGTTCGTTAAA

Supplementary Figure S1: Confirmation of the plasmid-encoded capsule expressing genes in *B. anthracis*

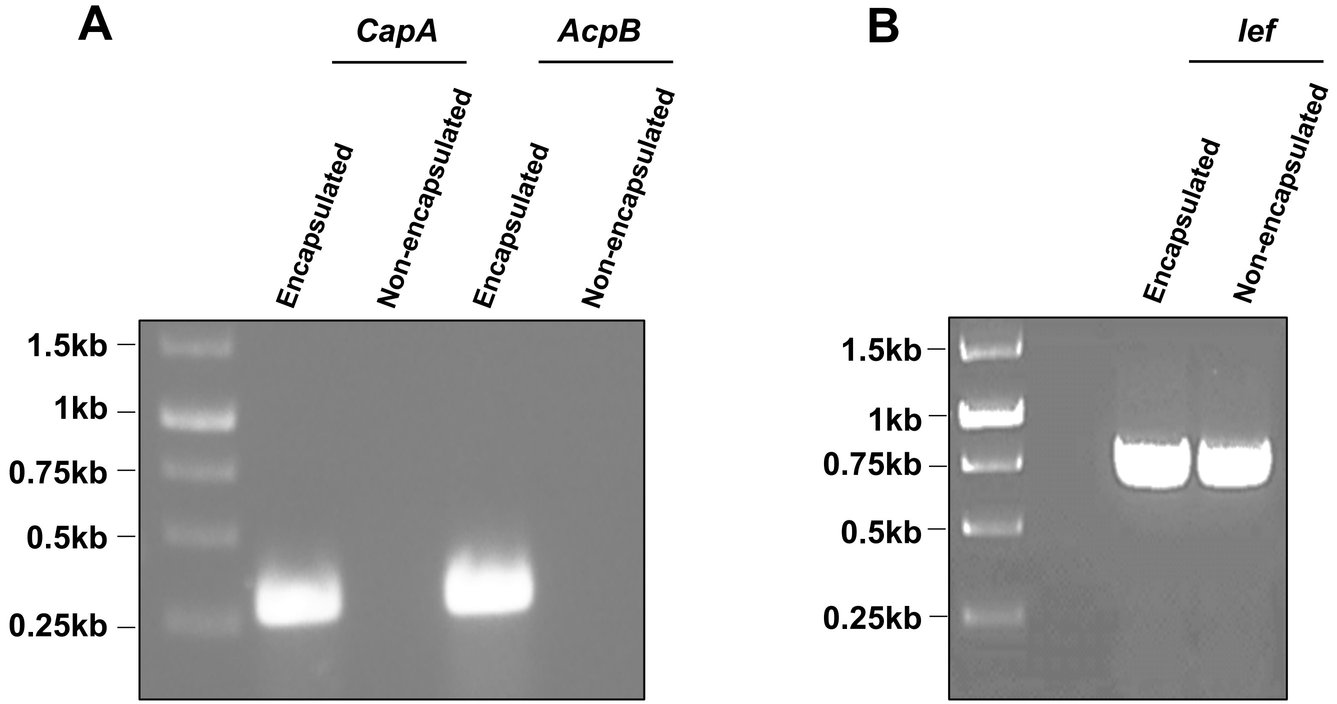

Figure S1: **Confirmation of the plasmid-encoded capsule expressing genes in *B. anthracis.*** Plasmid DNA from encapsulated and non-capsulated *B. anthracis* strains were used as templates for PCR amplification of the pXO2 and pXO1 plasmid encoded specific genes. **(A)** 300 kb amplicons for both pXO2 plasmid encoded genes, *CapA* and *AcpB.* were detected only in the encapsulated strains. **(B)** pXO1 plasmid encoded gene *lef* was amplified from both the encapsulated and non-capsulated strains.

Supplementary Figure S2: Loading controls for Immunoblot assay

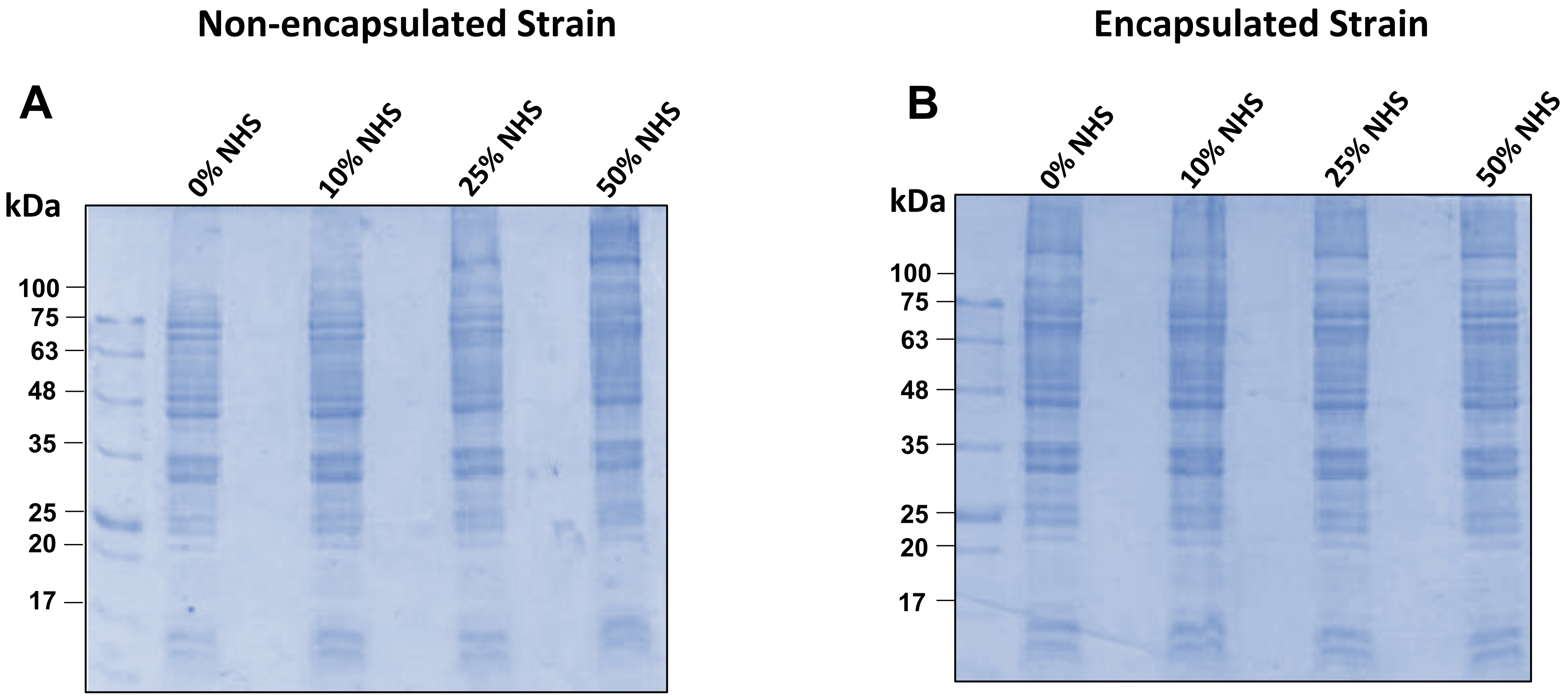

Figure S2: Loading control for immunoblot assays demonstrating serum concentration dependent binding of C3b and IgG on non-encapsulated and encapsulated strains of *B. anthracis*. Both bacterial strains were incubated with increasing concentration of normal human serum (NHS). Equal volume of bacterial lysates (representing 10^8 bacteria), as used for the immunoblot assay were added in each lane of SDS-PAGE and stained with coomassie brilliant blue G-250. SDS-PAGE profile for bacterial lysate incubated with different NHS concentration appears to be similar for both non-encapsulated strain **(A)** and encapsulated strains **(B)** of *B. anthracis*.

Supplementary Figure S3: Percentage of bacteria positive for C3b binding (Suppl. Data for Figure 2A)

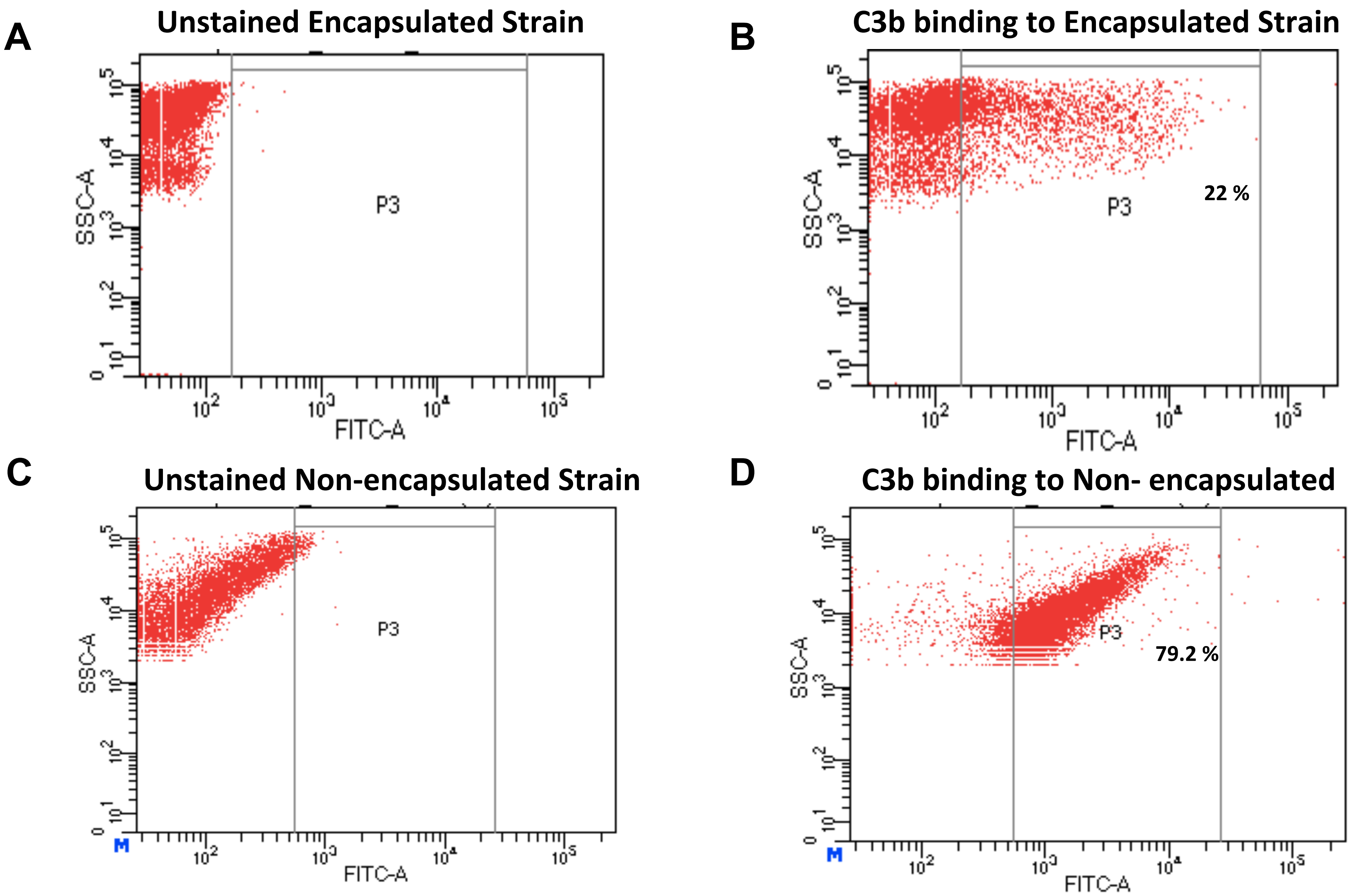

Figure S3: Flow cytometry scatter plot representing the percentage population positive for binding of C3b. Serum incubated bacterial cells probed with secondary antibodies were used as negative controls for both bacterial strains **(A & C).** A larger population of bacterial cells of the non-encapsulated *B. anthracis* strain were positive for C3b binding (79.2%) **(D)** in comparison to the encapsulated bacterial strain (22%) **(B).**

Supplementary Figure S4: Percentage of bacteria positive for CRP binding (Suppl. Data for Figure 5A)

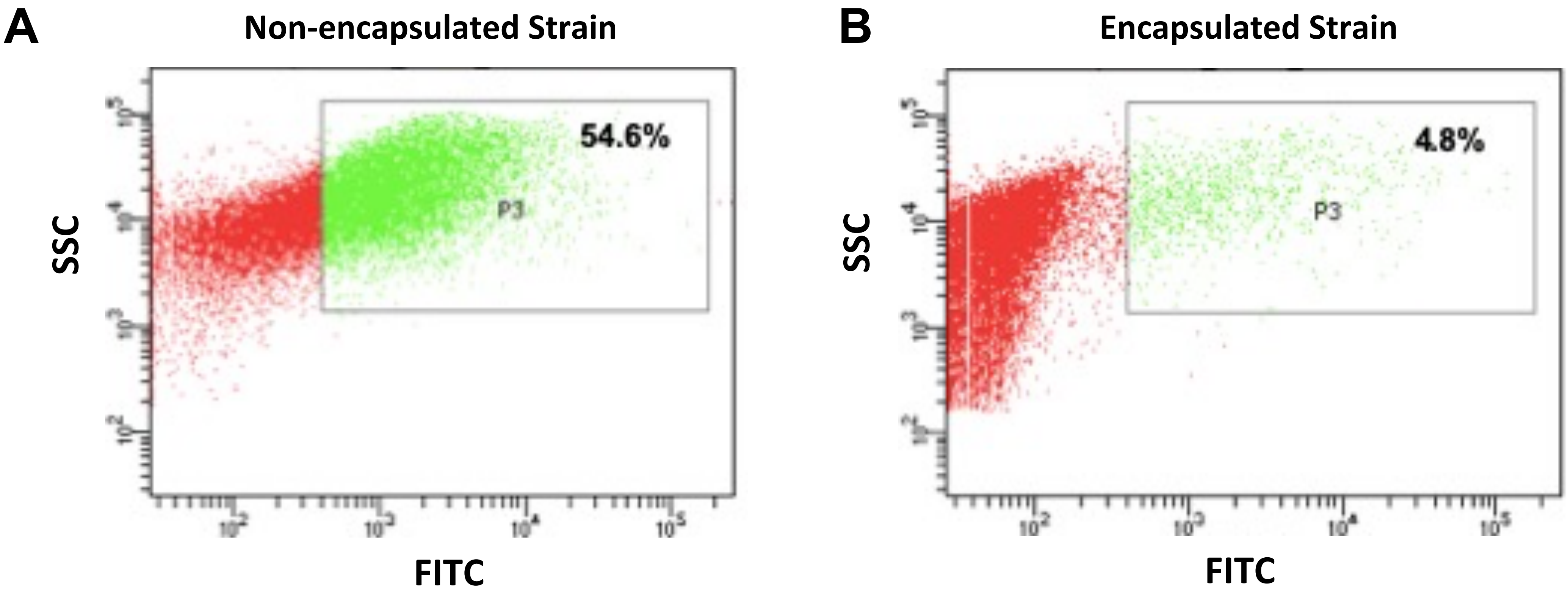

Figure S4: Flow cytometry scatter plot representing the percentage population positive for binding of CRP. Serum incubated bacterial cells probed with secondary antibodies were used as negative controls for both bacterial strains A larger population of bacterial cells of the non-encapsulated strain were positive for CRP binding (54.6%) **(A)** in comparison to the encapsulated bacterial strain (4.8%) **(B)**

Supplementary Figure S5: Percentage of positive bacteria for SAP binding (Suppl. Data for Figure 5B)

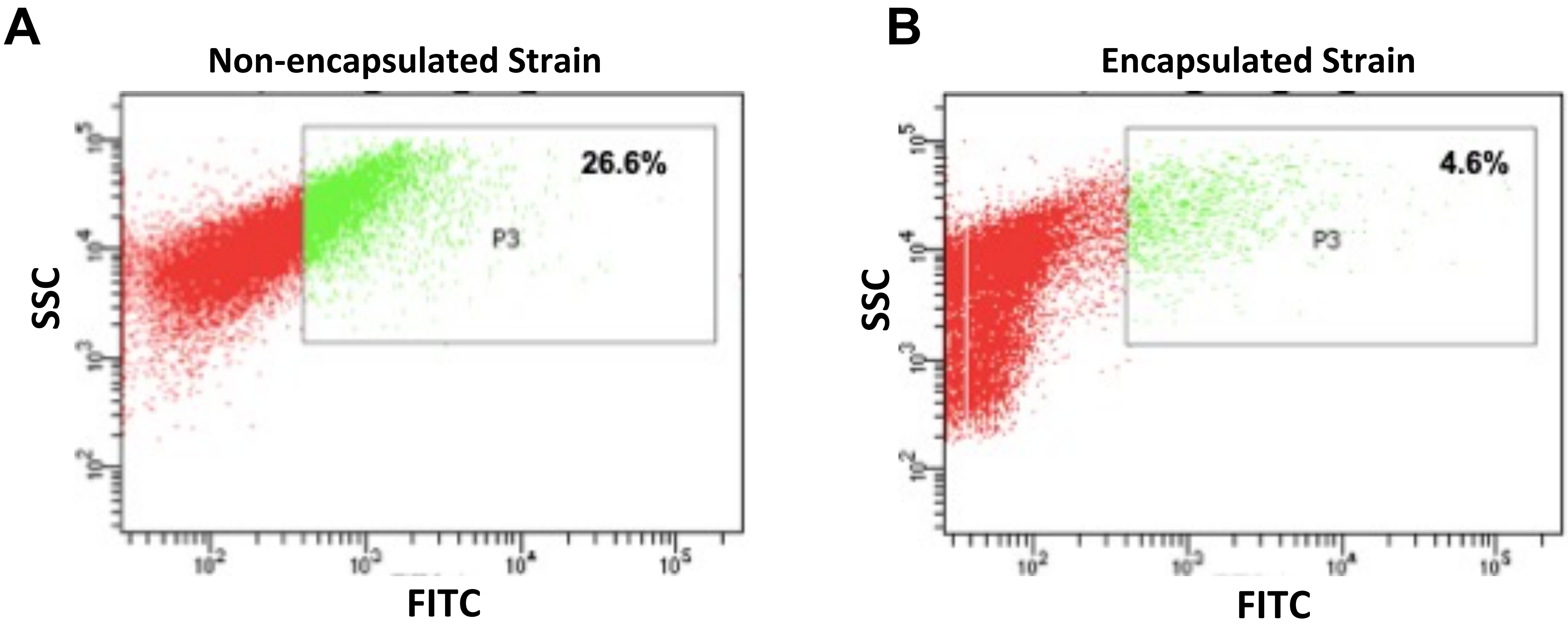

Figure S5: Flow cytometry scatter plot representing the percentage population positive for binding of SAP. Serum incubated bacterial cells probed with secondary antibodies were used as negative controls for both bacterial strains**.** A larger population of bacterial cells of the non-encapsulated strain were positive for CRP binding (26.6%) **(A)** in comparison to the encapsulated bacterial strain (4.6%) **(B).**

Supplementary Figure : Figure S6: Flow Cytometry histograms for C4BP and Factor H binding

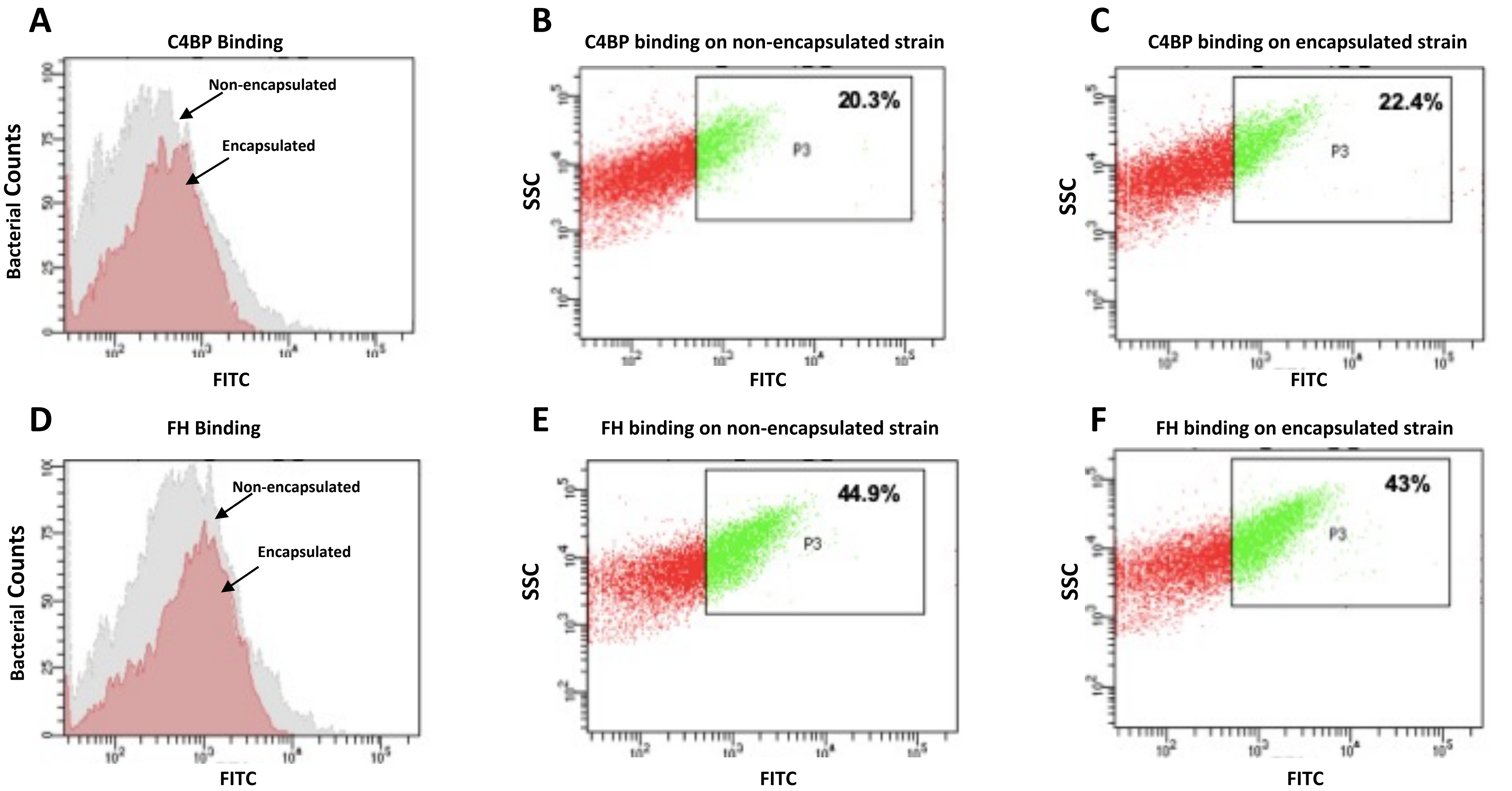

Figure S6: Flow cytometry histograms representing the binding of complement regulators C4BP **(A)** and Factor H **(D)** on encapsulated (red shade) and non-encapsulated (grey shade) *B. anthracis* strains. The binding of the complement regulators with the bacterial cells were detected by anti-human C4BP and anti-human Factor H antibodies. Percent positive population for C4BP binding with non-encapsulated **(B)** and encapsulated **(C)** *B. anthracis* strains. Percent positive population for FH binding with non-encapsulated **(B)** and encapsulated **(C)** *B. anthracis* strains. No significant difference was observed in the binding of C4BP & FH between the encapsulated and non-encapsulated strains
